# Significant Hypo-Responsiveness to GPVI and CLEC-2 Agonists in Pre-Term and Full-Term Neonatal Platelets and following Immune Thrombocytopenia

**DOI:** 10.1055/s-0038-1646924

**Published:** 2018-04-25

**Authors:** Alexander T. Hardy, Verónica Palma-Barqueros, Stephanie K. Watson, Jean-Daniel Malcor, Johannes A. Eble, Elizabeth E. Gardiner, José E. Blanco, Rafael Guijarro-Campillo, Juan L. Delgado, María L. Lozano, Raúl Teruel-Montoya, Vicente Vicente, Steve P. Watson, José Rivera, Francisca Ferrer-Marín

**Affiliations:** 1 Institute of Cardiovascular Science, IBR Building, College of Medical and Dental Sciences, University of Birmingham, Birmingham, United Kingdom; 2 Servicio de Hematología y Oncología Médica, Hospital Universitario Morales Meseguer, Centro Regional de Hemodonación, Universidad de Murcia, IMIB-Arrixaca, U765-CIBERER, Murcia, Spain; 3 Department of Biochemistry, University of Cambridge, Downing Site, Cambridge, United Kingdom; 4 Institute of Physiological Chemistry and Pathobiochemistry, University of Münster, Münster, Germany; 5 ACRF Department of Cancer Biology and Therapeutics, John Curtin School of Medical Research, Australian National University, Canberra, Australia; 6 Departamento de Ginecología y Obstetricia, Hospital Clínico Universitario Virgen de la Arrixaca. IMIB-Arrixaca, Murcia, Spain; 7 Centre of Membrane Proteins and Receptors (COMPARE), Universities of Birmingham and Nottingham, Midlands, United Kingdom; 8 Grado de Medicina, Universidad Católica San Antonio de Murcia, Murcia, Spain

**Keywords:** premature, full-term neonates, platelet hypo-responsiveness, ITAM-containing receptors, development, immune-induced thrombocytopenia

## Abstract

Neonatal platelets are hypo-reactive to the tyrosine kinase-linked receptor agonist collagen. Here, we have investigated whether the hypo-responsiveness is related to altered levels of glycoprotein VI (GPVI) and integrin α2β1, or to defects in downstream signalling events by comparison to platelet activation by C-type lectin-like receptor 2 (CLEC-2). GPVI and CLEC-2 activate a Src- and Syk-dependent signalling pathway upstream of phospholipase C (PLC) γ2. Phosphorylation of a conserved YxxL sequence known as a (hemi) immunotyrosine-based-activation-motif (ITAM) in both receptors is critical for Syk activation. Platelets from human pre-term and full-term neonates display mildly reduced expression of GPVI and CLEC-2, as well as integrin αIIbβ3, accounted for at the transcriptional level. They are also hypo-responsive to the two ITAM receptors, as shown by measurement of integrin αIIbβ3 activation, P-selectin expression and Syk and PLCγ2 phosphorylation. Mouse platelets are also hypo-responsive to GPVI and CLEC-2 from late gestation to 2 weeks of age, as determined by measurement of integrin αIIbβ3 activation. In contrast, the response to G protein-coupled receptor agonists was only mildly reduced and in some cases not altered in neonatal platelets of both species. A reduction in response to GPVI and CLEC-2, but not protease-activated receptor 4 (PAR-4) peptide, was also observed in adult mouse platelets following immune thrombocytopenia, whereas receptor expression was not impaired. Our results demonstrate developmental differences in platelet responsiveness to GPVI and CLEC-2, and also following immune platelet depletion leading to reduced Syk activation. The rapid generation of platelets during development or following platelet depletion is achieved at the expense of signalling by ITAM-coupled receptors.

## Introduction

Platelets are anuclear haematopoietic cells that play an essential role in haemostasis and its pathological counterpart thrombosis. Platelets also play critical roles in other physiological and pathological processes including inflammation, 1 infection, 2 vascular integrity, 3 development 4 and cancer metastasis. 5 Currently, we have a rudimentary understanding of the role of platelets in many of these functions and how these processes vary during development and throughout adulthood.

Thrombopoiesis takes place in multiple sites during development, beginning in the yolk sac before moving to the liver and finally to the bone marrow and spleen. 6 7 8 This means that, throughout development, circulating platelets are derived from more than one haematopoietic site. It is presently unclear to what extent this influences platelet function.

It is widely acknowledged that platelet reactivity is reduced in neonates, but the molecular basis underlying this is not known; the degree of hypo-reactivity and the extent to which this varies between agonists is also uncertain. 9 10 11 12 This is partly due to methodological issues related to the low volumes of blood available for experimentation, with many studies limited to single concentrations or small panels of agonists. 9 10 11 12 One consistent feature is a marked reduction in responsiveness to collagen. 13 14 Collagen activates Src and Syk tyrosine kinases downstream of the glycoprotein VI (GPVI)-Fc receptor γ-chain (FcRγ) complex, culminating in activation of PLCγ2. 15 Collagen also binds to integrin α2β1 which supports adhesion and net binding to GPVI. 15 However, no difference in α2β1 expression between neonatal and adult platelets has been reported, 10 13 with the reduced response to collagen being attributed to a reduction in phosphoinositide hydrolysis and Ca ^2+^ mobilization suggesting a defect in collagen signalling. 13 16

There are also reports of impaired responses to G protein-coupled receptor (GPCR) agonists, although this has not been seen in all cases, and several mechanisms have been proposed. The reduced response to adrenaline and thrombin has been attributed to decreased receptor expression, and in the case of thromboxane A _2_ mimetic U46619, to defective G protein-coupled activity. 10 17 18

Platelets from pre-term infants are also hypo-reactive in comparison to their full-term counterparts. 14 19 In the western world, more than 10% of babies are pre-term 20 and this population displays the highest incidence of intra-ventricular haemorrhage (IVH). IVH affects up to 25% of infants born with weights of less than 1,500 g and usually occurs in the first week of life. 11 The increase in risk of bleeding coinciding with the time of marked platelet hypo-reactivity raises the question of whether this contributes to the increase in IVH. 21 22

In this study, we have assessed the reactivity of human platelets from pre- and full-term neonates and that of mice platelets during late gestation and in neonates to collagen-related peptide (CRP) and to the snake venom toxin rhodocytin which activate GPVI and C-type lectin-like receptor 2 (CLEC-2), respectively. 23 Since CLEC-2 signals through a similar pathway to GPVI, a decrease in responsiveness to CLEC-2 could reflect a reduction in immunotyrosine-based-activation-motif (ITAM) signalling, rather than a specific loss of response to GPVI. Improving our understanding of the underlying mechanisms of platelet hypo-reactivity in foetal and neonatal life, will help to define the contribution of platelet hypo-reactivity in neonatal bleeding disorders and guide clinical decision making and management of haemostasis and thrombosis complications especially in the case of pre-term neonates.

## Materials and Methods

### Materials

The platelet glycoprotein screen assay kit for analysis of human GPIIIa (β3-integrin subunit), GPIbα and GPIa and the platelet calibrator kit for analysis of GPVI and CLEC-2 were from Biocytex (Marseille, France). Antibodies for the platelet calibrator kit were as follows: GPVI-mAb 1G5 24 ; α-CLEC-2-mAb AYP1 24 ; α-CD41a*APC; and α-CD62*PE was from BD Biosciences (Madrid, Spain); and Fibrinogen*Alexa Fluor 488 was from Life Technologies (Madrid, Spain). Protease-activated receptor 1 (PAR-1) peptide (TFLLR) and phorbol 12-myristate 13-acetate (PMA) were from Sigma-Aldrich (Madrid, Spain), and Human PAR-4 peptide (AYPGKF) was from Alta Biosciences (Birmingham, UK). Integrilin (eptifibatide) was from Glaxosmithkline (Middlesex, UK). Sodium dodecyl sulphate-polyacrylamide gel electrophoresis (SDS-PAGE) gels, polyvinylidene difluoride (PVDF) membranes, peroxidase conjugated secondary antibodies and enhanced chemiluminescence (ECL) mix were from GE Healthcare (Madrid, Spain). α-PLCγ2 (B-10), α-Syk (4D10) and anti-Fcγ chain (sc-390222, FcεRIγ [E-12]) were from Santa Cruz (Heidelberg, Germany). α-PLCγ2-Y1217 and α-Syk-525/526 were from Cell Signaling Technology (Leiden, The Netherlands). α-βActin was from Sigma-Aldrich and α-GPVI was from Abcam (Cambridge, UK). Anti-αIIb antibody 132.1 was a gift from Z.M. Ruggeri (Scripps Clinic, La Jolla, California, United States). For assays in mice, the antibodies were as follows: The mouse CLEC-2 monoclonal antibody (mAb) 17D9 and α-IgG2b*FITC were purchased from Bio-Rad (Hemel Hempstead, UK). α-P-Selectin*PE was from Novus Biologicals (Abingdon, UK). α-CD41*APC and α-CD41*PE were from eBioscience (Hatfield, UK) and BD Pharmigen (Oxford, UK), respectively. α-CD41*FITC, α-CD42b*FITC, α-CD49b*FITC, α-GPVI*FITC and α-IgG*FITC were from Emfret (Eibelstadt, Germany). α **-** GPIbα and immunoglobulin G (IgG) control used for immune depletion were also from Emfret. α-CLEC-2*FITC was from AbD Serotec (Kidlington, UK). Goat α-Rat*488 secondary antibody was from Fischer Scientific (Loughborough, UK). Other reagents were from recognized suppliers and of the highest analytical grade commercially available.

### Human Blood Collection

Cord blood (CB) samples were collected from healthy pre-term (26–34 gestational weeks) and full-term neonates (≥ 37 gestational weeks), admitted to the maternal-fetal unit of the University Hospital Virgen de la Arrixaca, Murcia, Spain. All neonates were born from uncomplicated pregnancies and had a normal platelet count (> 150,000 platelets/μL). Neonates were excluded from the study if mothers had a history of diabetes, hypertension, pre-eclampsia, active infection, drug or alcohol abuse, had taken aspirin during the 10 days prior to the delivery or there was a family history of abnormal haemostasis or any congenital disorder. Peripheral blood (PB) samples were collected from the antecubital veins of healthy adult volunteers who had not taken any medications during the 10 days prior to the study. All samples (CB and PB) were drawn in to 3.2% buffered sodium citrate tubes (Vacutainer System; Diagnostica Stago Becton Dickinson, Plymouth, UK). The study on humans was approved by the Ethics Committee of the Arrixaca University Hospital and followed the Helsinki Declaration. All adult volunteers and the parents of neonates provided written informed consent.

### Murine Blood Collection

Mouse embryos were dissected from pregnant females after maternal cervical dislocation, before being decapitated and allowed to bleed into 10 units/mL of heparin in phosphate-buffered saline (PBS). Neonatal mice were culled via intra-peritoneal injection of Euthatal (50–100 µL) before being decapitated and allowed to bleed into 10 units/mL of heparin. Where stated, adult mice were culled as for neonates to enable comparison of results of platelets prepared in the same way. Otherwise, adult mice were anaesthetised with isoflurane before CO _2_ narcosis, descending vena cava isolation and subsequent venepuncture collection.

For repeat blood collection from immune-induced thrombocytopenic mice, the animals were restrained and blood was withdrawn from the tail vein via needle-prick into 10 units/mL heparin; terminal bleeds were performed under isoflurane/CO _2_ as described above. All animal work was performed with U.K. Home Office approval under license PPL70/8286.

### Receptors Levels and Platelet Activation in Human Platelets

Platelet receptor expression in adult, pre-term and full-term blood samples was assessed by flow cytometry using a BD Accuri C6 flow cytometer device (Ann Arbor, Michigan, United States). The GP Screen and Platelet Calibrator kits (Biocytex), with the appropriate antibodies as stated above, were used following the manufacturer's instructions.

Adult blood and CB samples from both pre-term and full-term neonates were also assessed for agonist-induced surface P-selectin exposure and fluorescent fibrinogen binding to αIIbβ3 by flow cytometry using Accuri C6. Briefly, diluted blood was incubated under static conditions (30 minutes at room temperature) with PBS, as control for non-stimulated platelets, PAR-1 peptide (25 µM), PAR-4 peptide (250 µM), adenosine diphosphate (ADP) (25 µM), PMA (100 nM), CRP (5 µg/mL) and rhodocytin (100 nM) in the presence of α-CD41a*APC, as platelet marker antibody, α-CD62*PE and fibrinogen*Alexa Fluor 488. Reactions were terminated by 4% paraformaldehyde (PFA) (v/v) and subsequent 10-fold dilution with PBS. For each sample, run up to 10,000 platelets were identified and analysed by gating events on both CD41a*APC positivity and forward scatter-side scatter (FSC-SSC). Results were expressed as percentage of positively stained cell for P-selectin or fibrinogen, as compared with non-stimulated cells.

### Assessment of GPVI and CLEC-2 Signalling Pathway in Human Platelets by Immunoblotting

Human adult and full-term neonates' washed platelets in modified Tyrode's buffer were obtained as previously described. 25 For protein phosphorylation studies, washed platelets (6 × 10 ^8^ /mL) were stimulated under stirring conditions for 180 seconds with CRP (1–10 µg/mL) or rhodocytin (30–100 nM), using an aggregometer set at 37°C (Aggrecorder II Menarini Diagnostics, Florence, Italy). Eptifibatide (1 µM) was included to prevent platelet aggregation and stimulation was stopped by addition of SDS reducing sample buffer. Proteins in whole cell lysates were separated by SDS-PAGE and transferred to PVDF membranes by means of semi-dry transfer units. Blots were stepwise incubated with appropriate primary antibodies and as peroxidase-conjugated secondary antibodies (see the “Materials” section), and proteins were detected by chemiluminescence.

### Gene Expression Analysis of *GP6* , *CLEC1B* , *SYK* and *PLCG2* in Human Platelets

Total ribonucleic acid (RNA) was isolated from ultrapure platelets as we have recently reported in detail. 26 Retrotranscription reaction was performed using 35.2 ng of total RNA, according to the manufacturer's instructions (SuperScript III First Strand, Thermo Fisher Scientific). Gene expression was quantified on a LC480 real-time polymerase chain reaction (PCR) system (Roche Pharma, Basel, Switzerland) using Taqman Premix Ex Taq (Takara Bio Inc.) and a commercial probe for *GP6* (Hs_00212574), *FCER1G* (Hs_Hs00175408), *CLEC1B* (Hs_00212925), *SYK* (Hs_00895377), *PLCG2* (Hs01101857_m1) and *ACTB* (Hs_99999903).

### Murine Platelet Receptor Expression and Platelet Activation

To evaluate murine platelet receptor expression, diluted whole blood (10–20 × 10 ^9^ platelets/L) was stained (30 minutes at room temperature) with both α-CD41*APC and a fluorescein isothiocyanate (FITC)-labelled antibody (see above) specific to the target receptor: GPIbα, αIIbβ3, α2β1, GPVI and CLEC-2. Reactions were terminated with 4% PFA solution and 10-fold PBS dilution as above, and platelets acquired and analysed with the Accuri C6 software. Results were expressed as mean fluorescence of positive cells. Agonist-induced platelet activation in mice at known pre- and post-natal ages was evaluated, as in the human studies above, by flow cytometric analysis of fibrinogen-binding and P-selectin exposure in PBS-diluted murine blood (10–20 × 10 ^9^ platelets/L). Agonists used in these assays include PBS, as control for non-stimulated platelets, PAR-4 peptide (50, 100 and 250 µM), CRP (1, 5 and 10 µg/mL) and rhodocytin (10, 30 and 100 nM). Results were expressed as percentage of murine platelets positively stained for P-selectin or fibrinogen, as compared with non-stimulated cells.

### Murine Recovery from Immune-Mediated Thrombocytopenia

Immune thrombocytopenia was induced in adult mice by injection of 1.5 µg/g α-GPIbα, which caused a sharp and rapid decline in platelet count. 27 Control mice were injected with IgG control. Blood samples were collected before injection and daily post-injection for analysis of platelet receptor expression and dose–response assays as described above.

### Statistical Analysis

All statistical analysis was performed using GraphPad Prism V7.00 (California, United States). Differences between neonates and adults in platelet receptor levels, gene expression levels or platelet functional responses were assessed by *t-* test or Mann–Whitney U test as appropriated. For murine receptor studies, an ordinary one-way analysis of variance (ANOVA) was performed with a post hoc Dunnet's multiple comparisons test. For murine neonatal and post-thrombocytopenia functional assays, a two-way ANOVA was performed with a Dunnet's multiple comparisons post hoc test. For murine post-thrombocytopenia receptor assays, a two-way ANOVA was performed with a Sidak's multiple comparisons post hoc test. Significance was assumed with a *p* -value of ≤ 0.05 (*) or ≤ 0.005 (**).

## Results

### Platelets from Pre-Term and Full-Term Neonates Display Reduced Expression and Function of GPVI and CLEC-2

**Fig. 1:**
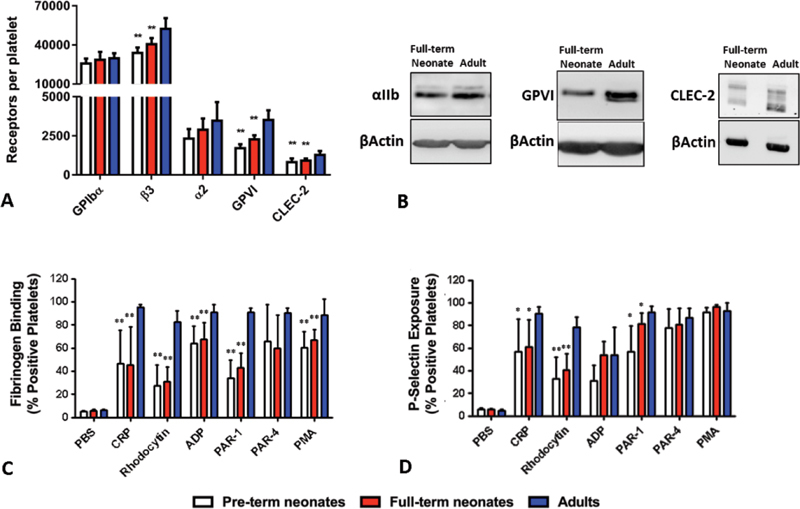
Flow cytometric assessment of receptor expression and function in platelets from premature, full-term neonates and adults. Platelet receptor expression was measured by ( **A** ) flow cytometry using Biocytex kits in human pre-term (white bars; *n* = 5) and full-term (red bars; *n* = 8–11) neonates and in adults (blue bars; *n* = 8–11), and by ( **B** ) western blotting, as described in the “Material and Methods” section. Western blots images are representative of assays in platelet lysates from different full-term neonates ( *n* = 6) and adults ( *n* = 6). In activation experiments, platelets from neonates (white and red bars; *n* = 5 and *n* = 6, respectively) and adults (blue; *n* = 6) were activated by collagen-related peptide (CRP) (5 μg/mL), rhodocytin (100 nM), adenosine diphosphate (ADP) (25 µM), Protease-activated receptor (PAR)-1 (25 µM) and PAR-4 (250 µM) peptides, and phorbol 12-myristate 13-acetate (PMA) (100 nM). The binding of fluorescently labelled fibrinogen ( **C** ) and the P-selectin exposure ( **D** ), was monitored for 30 minutes at room temperature (RT), post-agonist stimulation. Values are mean plus standard deviation. * and ** denote *p* ≤  0.05 and *p* ≤  0.005, respectively, versus adults.

We explored the mean platelet volume and expression levels of human platelet receptors in pre-term neonates (median gestational age of 32.3 [range, 29.8–34.4] weeks), full-term neonates and adults. The mean platelet volume was similar in all three groups ( Supplementary Fig. S1A , available in the online version). In contrast, there was a significant (30–35%) reduction in the levels of GPVI and CLEC-2 in pre-term and full-term neonates versus adults as shown by flow cytometry ( Fig. 1A ) and by immunoblotting ( Fig. 1B ). In agreement with the above findings, quantitative reverse transcription PCR (qRT-PCR) experiments showed that neonatal platelets displayed a significant reduction in *GP6* (60%) and a mild decrease in *CLEC1B* (30%) messenger RNA (mRNA) levels, compared with adult platelets ( Supplementary Fig. S2A , B , available in the online version). In line with the decrease in GPVI, the expression of Fcγ chain, which associated with GPVI to form a functional collagen immunoreceptor, 28 29 was also reduced at protein and mRNA level in neonatal platelets ( Supplementary Fig. S3A–C , available in the online version). In addition, and in concordance with previous studies, 10 pre-term and full-term neonates displayed a mild reduction in expression of integrin αIIbβ3, but no significant change in the levels of integrin α2β1 and GPIbα ( Fig. 1A ).

**Fig. 2:**
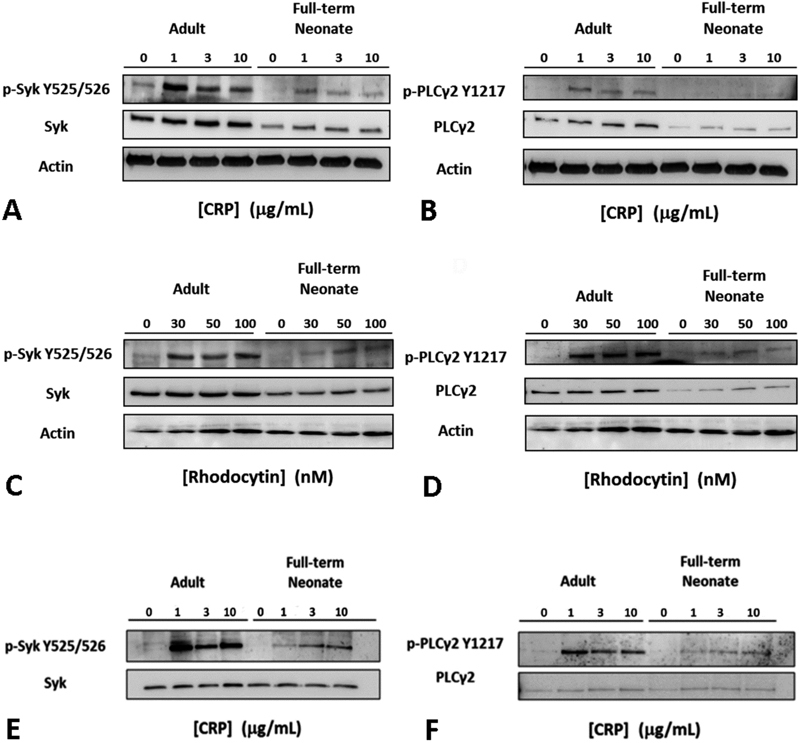
Expression and phosphorylation of Syk and PLCγ2 in platelets from adult and full-term neonates in response to collagen-related peptide (CRP) and rhodocytin. Human washed platelets were diluted to 600 × 10 ^9^ platelets/L and stimulated with different dose of CRP ( **A** , **B** ; **E** , **F** ) and rhodocytin ( **C** , **D** ) for 5 minutes under stirring conditions. Reactions were stopped by adding one volume of 5× reducing sample buffer. In panels **E** and **F** , identical amounts of total Syk ( **E** ) and PLCγ2 ( **F** ) were loaded in the gel. Samples were developed by sodium dodecyl sulphate-polyacrylamide gel electrophoresis (SDS-PAGE) and analysed using phospho-specific antibodies. The images shown are representative of 4 ( **A** – **D** ) and 3 ( **E** , **F** ) experiments with independent platelet samples.

**Fig. 3:**
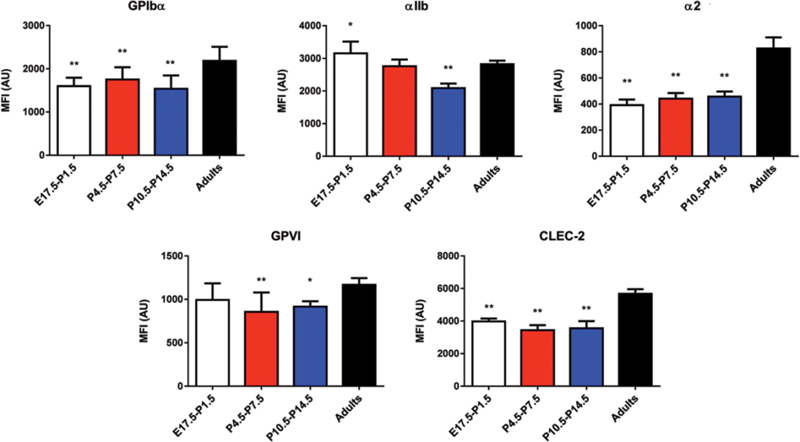
Receptor expression in mouse platelets during development. Platelet surface receptor expression profiles in mice platelets were assessed by flow cytometry with fluorescently labelled antibodies, as reported in the “Materials and Methods” section. Results are expressed as mean plus standard deviation of the median fluorescence of platelet populations in different mice (gestational and neonatal platelet, *n* = 12–26; adults platelets *n* = 6); * *p* ≤  0.05; ** *p* ≤  0.005

Developmental changes in human platelet reactivity were assessed in flow cytometric experiments by monitoring fibrinogen-binding and P-selectin expression following platelet stimulation. Compared with adults, platelets from pre-term and full-term neonates exhibited a marked reduction in fibrinogen binding in response to CRP, rhodocytin and PAR-1 (over 50%), along with a moderate decrease for ADP and PMA (∼25%) and no change in response to PAR-4 ( Fig. 1C ). Agonist-induced platelet expression of P-selectin, a marker of α-granule release, was also significantly decreased in pre-term versus adult platelets for CRP, rhodocytin and PAR-1. In full-term neonates, P-selectin exposure was markedly reduced in response to CRP and rhodocytin, with only a mild decrease in PAR-1 ( Fig. 1D ).

These results demonstrate a hypo-sensitivity to GPVI and CLEC-2 agonists, which is in part explained by a decrease in receptor expression.

### Expression and Tyrosine Phosphorylation of Syk and PLCγ2 is Reduced in Neonatal Platelets

To investigate whether ITAM receptor signalling varies during development and contributes to the defect in platelet activation by GPVI and CLEC-2, human platelets from full-term neonates and adults were compared for Syk and PLCγ2 expression, and for tyrosine phosphorylation of these key signalling proteins after stimulation with CRP and rhodocytin. There was a reduction in the total level of Syk and PLCγ2 proteins in resting neonatal platelets when compared with adult platelets (Syk: 0.53 ± 0.14 vs. 0.73 ± 0.19 arbitrary units; PLCγ2: 0.15 ± 0.1 vs. 0.27 ± 0.06 arbitrary units) as measured by western blotting ( Fig. 2 ). The gene expression level of SYK was also lower in platelets from full-term neonates versus adult platelets ( Supplementary Fig. S2C , available in the online version). The level of PLCγ2 mRNA was too low for quantitation. Stimulation with either CRP or rhodocytin for 5 minutes induced dose-dependent tyrosine phosphorylation of Syk and PLCγ2, as assessed with phospho-specific antibodies to the activation site in Syk (residues 525/526) 30 and to an established marker of PLCγ2 activation (Tyrosine 1217). 31 As illustrated in Fig. 2 , phosphorylation of Syk and PLCγ2 was markedly reduced in neonatal platelets at all concentrations of CRP ( Fig. 2A, B ) and rhodocytin ( Fig. 2C, D ). This reduced phosphorylation of Syk and PLCγ2 in neonatal platelets is still appreciable after normalization for the reduced level of expression of Syk and PLCγ2 ( Fig. 2E, F ), thus supporting the impairment in the signal transduction pathway common to GPVI and CLEC-2 in full-term neonates.

### Gestational and Neonatal Mice Platelets Display a Hypo-Reactivity to GPVI and CLEC-2

To further assess the influence of the gestational age on the change in platelet reactivity, we extended our research to mouse platelets (E17.5 to P14.5 and adult). As with human studies, we first measured the levels of GPVI, CLEC-2 and other major glycoproteins by flow cytometry. As with human platelets, a mild reduction in the levels of GPVI and CLEC-2 was observed in gestational and neonatal mice platelets relative to adults ( Fig. 3 ), which cannot be explained by a change in mean platelet volume ( Supplementary Fig. S1B , available in the online version).

**Fig. 4:**
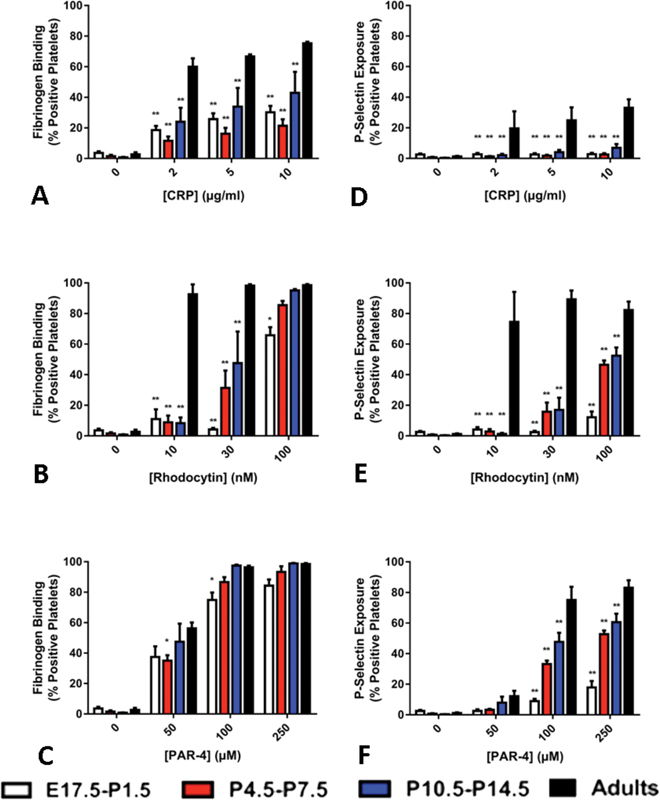
Agonist-induced activation of mouse platelets during development. Activation of mouse platelets during development was monitored by flow cytometry for ( **A** – **C** ) fluorescently labelled fibrinogen binding and ( **D** – **F** ) expression of P-selectin expression in response to ( **A** , **D** ) collagen-related peptide (CRP) (2, 5, 10 µg/mL); ( **B** , **E** ) rhodocytin (10, 30, 100 nM) and ( **C** , **F** ) protease-activated receptor (PAR)-4 peptide (50, 100, 250 µM). Values are mean plus standard error of the percentage of positive labelled platelets achieved in 3 to 9 different samples per group. * and ** denote *p* ≤  0.05 and *p* ≤  0.005, respectively, in comparison to values in adults platelets.

We performed functional studies on the murine platelets in response to CRP, rhodocytin and PAR-4 peptide. Foetal (E17.5) and early neonatal (P1.5–P7.5) mice platelets displayed a significant impairment in fibrinogen binding in response to all concentrations of CRP ( Fig. 4A ) and to low and moderate concentrations of rhodocytin ( Fig. 4B ). By 2 weeks of age (P10.5–P14.5), mice platelets stimulated with high concentrations of rhodocytin recovered their fibrinogen-binding capacity ( Fig. 4B ). In contrast, we observed only a mild reduction in fibrinogen binding of murine platelets aged E17.5 to P14.5 in response to PAR-4 peptide ( Fig. 4C ). A more severe decrease in response was observed in mice platelets for agonist-induced expression of P-selectin. Thus, as compared with adults, the surface expression of the α-granule protein was significantly reduced for all three stimuli in foetal and neonatal mice platelets, with recovery increasing with age ( Fig. 4D – F ).

These results demonstrate that as with human platelets, mouse platelets show impairment in response to GPVI and CLEC-2 in foetal, neonatal and early post-natal life. In the case for PAR-4, they also show a marked reduction for the expression of P-selectin, and a mild decrease in activation of αIIbβ3 in late gestational and neonatal platelets.

### Platelets are Hypo-Responsive to GPVI and CLEC-2 following Immune-Induced Thrombocytopenia

**Fig. 5:**
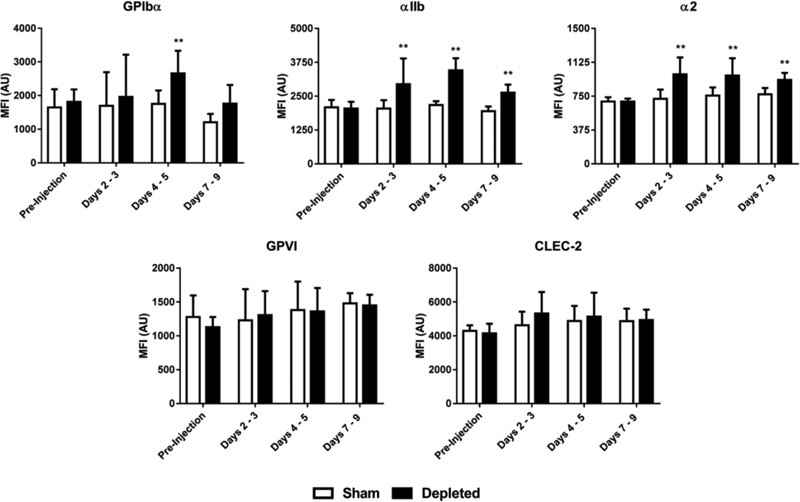
Receptor levels in mice platelets before and after immune depletion. Surface receptor expression profiles in mice were assessed following platelet immune depletion using fluorescently labelled antibodies as described in the Supplementary Methods. Data are shown as mean plus standard deviation of the median fluorescence of the platelet population achieved in 12 to 24 different samples in each group. ** denotes *p* ≤  0.005 versus values in mice treated with a control immunoglobulin G (IgG) antibody (sham).

We hypothesized that the hypo-responsiveness to GPVI and CLEC-2 may be related to the need to generate sufficient numbers of platelets to keep pace with the growth of embryos and neonates. To model this high-pressure platelet production environment in adult mice, we established an immune-induced thrombocytopenia mice model in which we assessed the platelet receptor levels and activation by GPVI, CLEC-2 and PAR-4. We observed that the new platelets generated following immune depletion have increased size ( Supplementary Fig. S1C , available in the online version). In concordance with this finding, the level of αIIbβ3 and α2β1 integrins increased slightly following immune depletion ( Fig. 5 ). In contrast, we observed no significant changes in the expression of the ITAM receptors, GPVI and CLEC-2, following immune depletion ( Fig. 5 ).

**Fig. 6:**
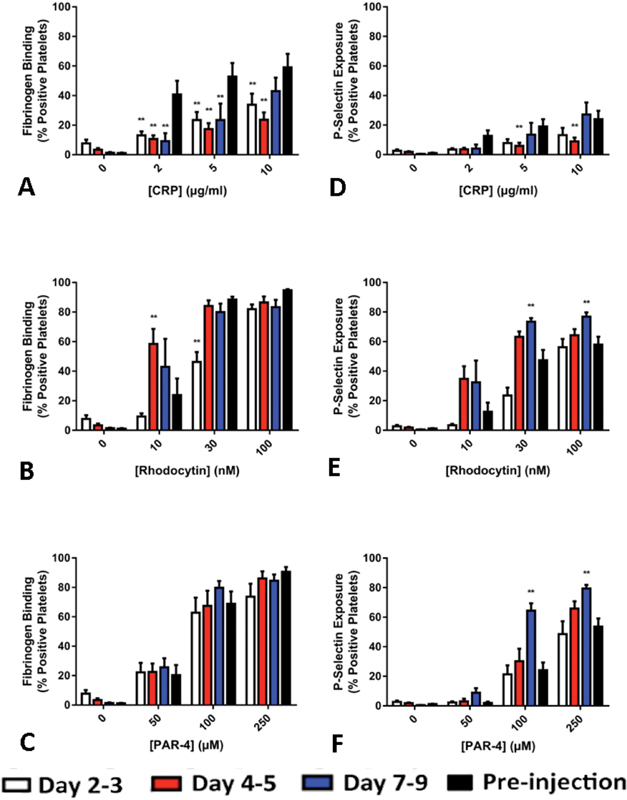
Measurement of murine platelet function following immune depletion. Mice were injected with α-GPIbα antibody to induce immune thrombocytopenia. Blood samples were collected before platelet depletion and thereafter for up to 9 days. Platelet reactivity was measured via ( **A** – **C** ) fluorescent fibrinogen binding and ( **D** – **F** ) P-selectin exposure in response to increasing doses of ( **A** , **D** ) collagen-related peptide (CRP), ( **B** , **E** ) rhodocytin and ( **C** , **F** ) protease-activated receptor (PAR)-4 peptide. Data are shown as mean plus standard error of the percentage of positive platelets achieved in 6 to 9 different samples in each group. ** denotes *p* ≤  0.005 versus values found in mice before injection of the α-GPIbα antibody.

The increase in fibrinogen-binding and P-selectin expression in response to CRP was markedly reduced in the first few days following immune thrombocytopenia ( Fig. 6A, D ). A similar but less severe pattern was observed in response to low-to-moderate concentrations of rhodocytin ( Fig. 6B , E ). By contrast, there was no decline in platelet activation induced by PAR-4 following immune thrombocytopenia ( Fig. 6C , F ).

## Discussion

Several studies have shown that neonatal platelets are hypo-responsive to various agonists such as ADP, epinephrine, thromboxane analogues, thrombin and collagen. This is characterized by decreased aggregation, secretion and expression of platelet activation upon stimulation. There is considerable variation among these studies on the degree of reduction in platelet reactivity, which most likely relates to differences in procedures for blood sampling, platelet function testing and limited sample volume. 10 11 12 19 32 Developmentally regulated expression of platelet receptors and receptor-coupled signal transduction can underlie the different reactivity of neonatal and adult platelets. 11 12 32 This study focused on the expression and function of the ITAM receptors GPVI and CLEC-2 during development in human and murine species, and in a murine model of haematopoietic stress. Hypo-responsiveness of neonatal platelets to collagen has been consistently seen in previous studies, and is more dramatic than that to GPCR agonists. 9 10 11 12 13 14

In agreement with previous reports, we observed developmental regulation of the expression of major adhesive platelet receptors in human (αIIbβ3; Fig. 1 ) 10 12 19 and mice (GPIbα, and αIIbβ3 and α2β1 integrins; Fig. 3 ). 33 We speculate that a moderate but significantly reduced level of integrin αIIbβ3 in platelets from pre-term and full-term neonates contributes to the mild impairment in αIIbβ3 activation (i.e. fibrinogen binding) by all agonists. In mice, however, αIIbβ3 levels in gestational and early post-natal life are similar to that in adults ( Fig. 3 ), while there is a marked impairment in fibrinogen binding in response to CRP, rhodocytin and, to a lesser extent, PAR-4. Thus, this defect may be mainly contributed by impairment in agonist-induced conformational changes and activation of the integrin, which was not evaluated in this study.

The major novel finding of our work is that during foetal and neonatal life platelet signalling through GPVI and CLEC-2 is impaired in mice and human platelets. While this investigation was underway, a study has also observed a reduced platelet response to CRP and rhodocytin in neonatal human platelets. 34 In addition to extending these findings to an earlier stage in human development (pre-term infants) and to development in mice, we also show, for the first time, that two mechanisms act synergistically to give rise to the impaired response to the two ITAM receptor agonists. The first one is a reduction in the level of GPVI, its associated protein Fcγ chain 28 29 and CLEC-2 in neonatal human platelets. This reduction is not accounted by significant changes in platelet volume ( Supplementary Fig. S1 , available in the online version), but is associated to changes at the transcriptional level, as indicated by reduced *GP6* , *FCER1G* and *CLEC1B* mRNA levels compared with adult platelets ( Supplementary Figs. S2 and S3 , available in the online version). Consistently, the level of these ITAM receptors is also reduced in gestational and neonatal murine platelets compared with adult platelets. In this study, we found no significant changes in platelet volume during mice development ( Supplementary Fig. S1 , available in the online version), contrarily to previous study. 7 This discrepancy is most likely due to analytical differences in blood collection and the device used to assess platelet volume.

The second mechanism contributing to the hypo-responsiveness of neonatal platelets to GPVI and CLEC-2 agonists is a regulation in development of the expression of key proteins in ITAM receptor signalling, such as Syk and PLCγ2, most likely at the transcriptional level as neonatal platelets display lower *SYK* mRNA levels. The mechanisms underlying transcriptional differences in genes of the ITAM receptor pathways remain unknown, and may include potential changes in the activity of factors involved in the regulation of *GP6* , *FCER1G* , *CLEC1B* and *SYK* .

Taken together, these two mechanisms contribute, in a degree that cannot be delineated from our study, to a significant impairment of signalling downstream of GPVI and CLEC-2, as reflected by a reduced Syk and PLCγ2 phosphorylation (this study), and the previously reported decrease in Ca ^2+^ mobilization in neonatal platelets in response to collagen. 16

Moreover, using a mouse model, we show that the responses to GPVI and CLEC-2 are also sharply reduced in adult mice within the first 3 days of recovery from immune thrombocytopenia, where a high thrombopoietic pressure would exist ( Fig. 6 ). This finding is in agreement with a very recent study showing that GPVI-ITAM signalling is partially inactive in newly formed platelets generated in response to acute thrombocytopenia. 35 Thus, it could be speculated that the rapid generation of platelets at the time of need (i.e. in development and recovery from a marked decrease in platelet count) may be due to induction of neonatal thrombopoiesis, thus resembling generation of red cells in response to acute anaemia which occurs through neonatal rather than adult erythropoiesis. 36 37 38 However, our current data suggest that the pattern of murine platelet reactivity following immune depletion in mice is complex and likely influenced by multiple factors. For instance, we observed an increased P-selectin exposure of days 7 to 9 platelets after depletion, in response to PAR-4 and rhodocytin. This is unlikely due to an increased platelet size, which is almost negligible at days 7 to 9 as compared with days 2 to 5 after depletion, and may be related to the rebound in platelet reactivity to these agonists. In addition, we found a puzzling increase in fibrinogen binding of days 4 to 5 platelets in response to 10 nM rhodocytin. In their recent study, Gupta et al 35 found that newly formed young platelets have increased reactivity to thrombin when immune depletion is achieved by an anti-GPIbα antibody, but not if this is induced by anti-αIIbβ3 antibody (day 5). They also found an unaltered response to rhodocytin (1 μg/mL) at day 5 following immune thrombocytopenia. Further studies are required to fully clarify the responsiveness of newly formed young platelets released after immune thrombocytopenia.

In contrast to the study of Baker-Groberg et al showing higher platelet-surface P-selectin expression in resting neonatal platelets, 34 we found that this expression was similar to that in resting adult platelets. The fact that we used adult venous blood and CB, whereas the previous study assayed capillary blood, 34 may account for the discrepancy. As in humans, in resting conditions, the platelet surface P-selectin levels were similar in murine neonatal and adult platelets. Moreover, the level of P-selectin expression was unaffected following immune thrombocytopenia. Notably, while in humans total P-selectin content is similar between adult and neonatal platelets, 39 in mice it has been suggested that P-selectin expression is developmentally regulated. 40 Thus, the marked reduction in P-selectin secretion induced by CRP and rhodocytin in gestational and early neonatal mice platelets that we have observed may be mediated by a combination of reduced activation of their ITAM receptors and by developmental up-regulation of the α-granule protein. In contrast, the impaired P-selectin response to PAR-4 may be due solely to up-regulation in development, as PAR-4-induced activation of fibrinogen binding is similar in gestational, neonatal and adult mice.

The functional significance of this decrease in ITAM receptor expression and signalling in platelet activation during ontogeny is currently unclear. The foetus has little threat of trauma during development other than the birth process, and GPVI has a minor role in haemostasis. 41 However, platelets are exposed to collagen during vasculogenesis and angiogenesis and a reduced responsiveness to GPVI may help to avoid unwanted thrombosis. In this context, it has recently been reported that infusion of adult platelets into mice at E14.5 leads to the rapid formation of occlusive thrombi, 42 and that transfusion of human adult platelets into neonatal blood leads to a hyper-coagulable profile. 43

To unravel the physiological relevance of reduced CLEC-2 levels during the first stages of the development, where it plays a key role in blood–lymphatic separation, further research will be required. Podoplanin expression changes during ontogeny 44 : in the first stages, podoplanin is highly and widely expressed, but over time its expression becomes restricted. 45 46 Importantly, podoplanin is strongly expressed in the villous stroma of the placenta, 47 and during angiogenesis of the foetal vessels, platelets could be exposed to the high amount of placenta podoplanin. Thus, reduction of platelet levels of CLEC-2 could be a compensatory mechanism to avoid excessive platelet activation during development and to safeguard neonatal haemostasis.

Our current results are of potential clinical significance, especially as premature human babies have an increased risk of IVH. 11 The pathogenesis of IVH in pre-term infants is complex and multifactorial, and the contribution of platelet hypo-reactivity is still unclear. 48 However, the poorly developed germinal matrix vessels in premature infants have been established as a contributing factor in the aetiology of IVH. 32 49 Recent studies in murine embryos have demonstrated that deficiency of CLEC-2 or podoplanin, its endogenous ligand, is associated with impaired angiogenesis and severe brain haemorrhaging. 4 During development, CLEC-2 induces platelet activation and subsequent formation of platelet aggregates through integrin αIIbβ3, to maintain the integrity of the nascence blood vessels in the brain. Thus, the reduced CLEC-2 and integrin αIIbβ3 levels in neonatal platelets that we report may contribute to the increase risk of IVH. Moreover, apart from considering body weight and surface on the newborns, new insights into the mechanisms of hypo-reactivity in neonatal platelets could aid the adjustment of anti-platelet therapy in neonates for the prevention of arterial thrombosis in conditions such as congenital heart disease, assist devices, prosthetic valves or systemic-to-pulmonary shunt implants. 50

There are several limitations of this study. The number of neonatal samples is relatively low due to limited availability and logistical difficulties. The use of CB as a source of neonatal platelets might not be ideal, but it is not otherwise possible to collect the volumes that were needed for all assays. Importantly, however, it has been reported that neonatal platelets from the cord and PB are similarly hypo-reactive. 19 In mice, the small sample volumes have limited the number of functional studies that could be performed.

In conclusion, in this study we show that platelet expression and signalling of GPVI and CLEC-2 are reduced in neonates of mice and humans. This hypo-reactivity of ITAM receptors may prevent unwanted platelet activation during vascular development, but an excessive ITAM signalling impairment could contribute to the increase in risk of IVH in neonates.

## Notes

**What is known about this topic?**

- Neonatal platelets display degrees of hypo-responsiveness to most platelet agonists, but most dramatically to collagen that activates platelets downstream of the ITAM containing receptor GPVI.
- Platelets also contain the hemi-ITAM receptor CLEC-2, which plays a critical role in blood–lymphatic separation in development.
- The potential contribution of developmental changes in ITAM receptor's expression or signalling in neonatal platelets' hypo-responsiveness is unknown.

**What does this paper add?**

- Pre-term and full-term neonates display mildly reduced platelet expression of ITAM receptors GPVI and CLEC-2, accounted for at the transcriptional level. They also show a pronounced ablated activation downstream of these receptors reflected in impaired integrin αIIbβ3 activation, P-selectin secretion and Syk and PLCγ2 phosphorylation.
- Mouse platelets are also hypo-responsive to GPVI and CLEC-2 from late gestation to 2 weeks of age, and following immune thrombocytopenia.
- Our study provides a mechanistic explanation for the hypo-responsiveness of neonatal platelets to collagen.

## Footnotes

*
*These authors contributed equally to the study.*
