# Supplementary material for: Significant Hypo-Responsiveness to GPVI and CLEC-2 Agonists in Pre-Term and Full-Term Neonatal Platelets and following Immune Thrombocytopenia
Source: Thromb Haemost. 2018 Apr 25;118(6):1009–20. doi: 10.1055/s-0038-1646924 (PMC6202930; doi:10.1055/s-0038-1646924)
Supplement: Supplementary file 1 — Supplementary Material [file 10-1055-s-0038-1646924-s180042.pdf]

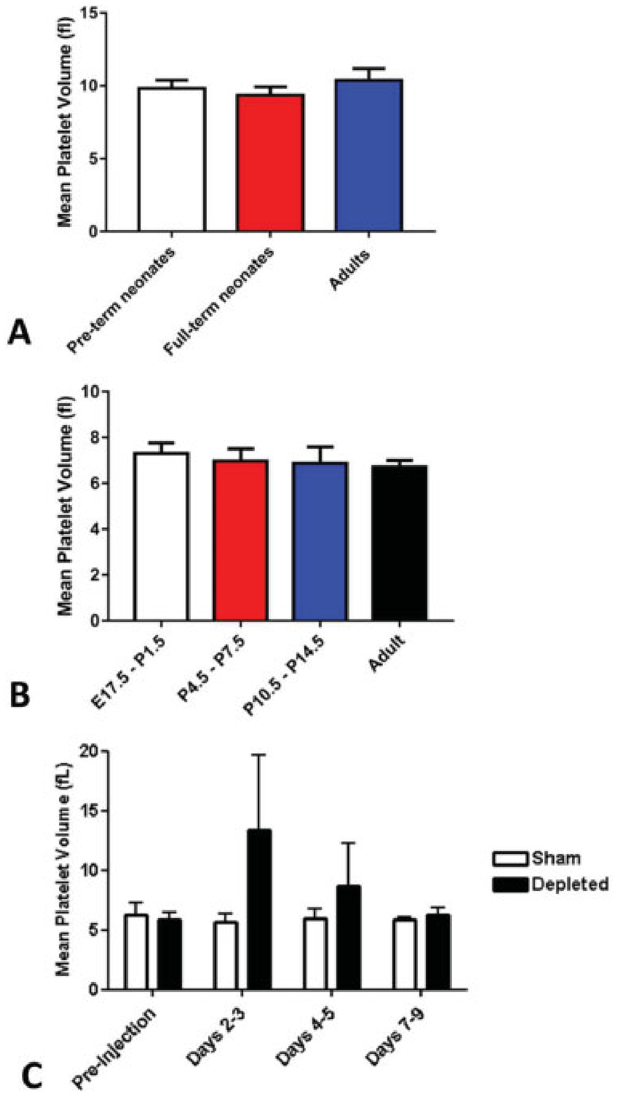

Fig. S1. Mean platelet volume in whole blood of human and mouse during development, and in mouse following immune-depletion. The mean platelet volume in cord blood samples from pre-term and full-term neonates and peripheral blood from adult was determined by means of a Sysmex haematological analyser (A). In mice studies, during development (B) and following injection or anti-GPIba or control IgG, as detailed in Material and Methods, mean platelet volume was measured in PBS diluted blood samples by means of an Abx Pentrablood analyzer. Results are show as mean values plus standard deviation (n = 3-10).

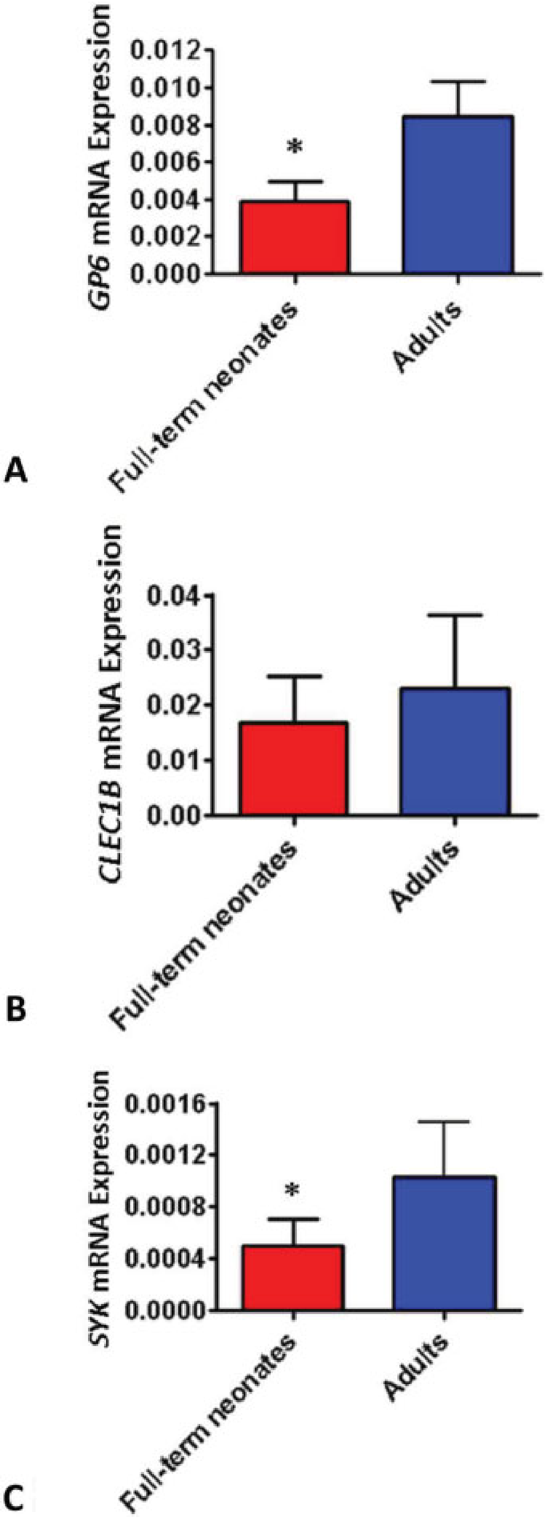

Fig. S2. Gene expression of *GP6* , *CLEC1B* and *SYK* in full-term and adult platelets. The mRNA expression levels of (A) GP6, (B) *CLEC1B* and (C) *SYK* were measured by quantitative real time (qRT)-PCR and normalized with respect to β-actin mRNA (*ACTB*). Data are show as mean plus standard deviation (n = 10). * denotes $p \leq 0.05$ vs. values in adults.

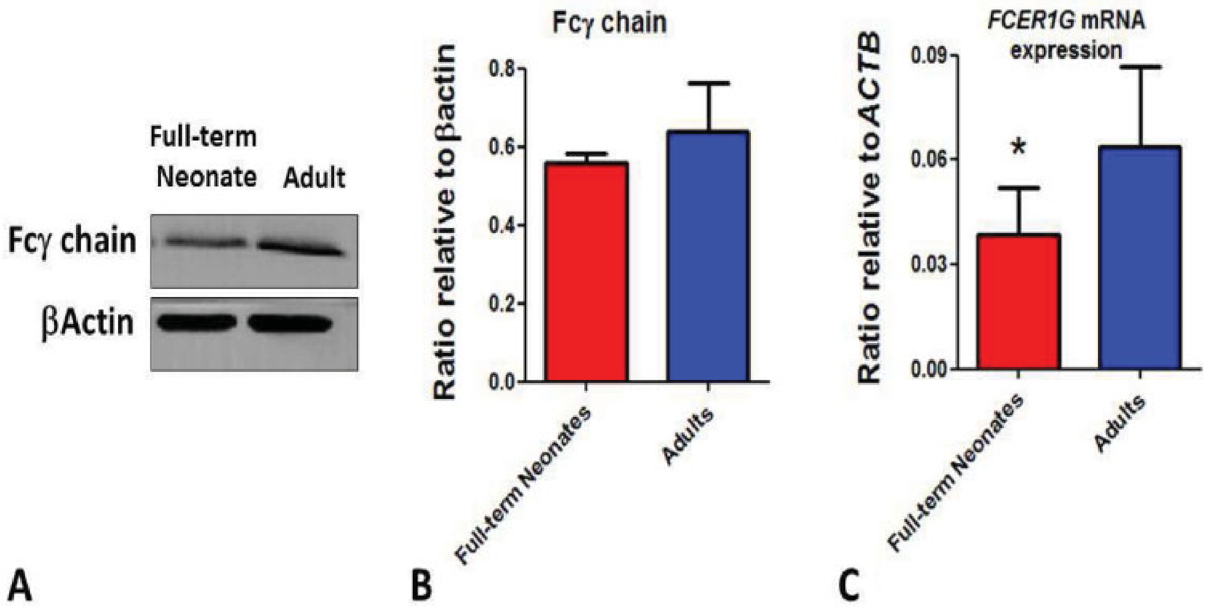

Fig. S3. Levels of Fcγ chain and *FCER1G* mRNA in platelets from full-term neonates and adults. Level ol Fcγ chain was measured in platelet lysates from full-term (red bars; n = 4) neonates and adults (blue bars; n = 4), by western blotting with anti-Fcγ chain monoclonal antibody (sc-390222, Santa Cruz) as described in Materials and Methods. A representative western blot image (A) and densitometry analysis of neoantes vs. adults (B) are displayed. In C) the mRNA expression levels of *FCER1G* were measured by quantitative real time (qRT)-PCR and normalized with respect to β-actin mRNA (ACTB). Data are show as mean plus standard deviation (n = 10). * denotes $p \leq 0.05$ vs. values in adults.
